# 782. Infectious Complications of Lung Transplant for COVID-Associated Lung Injury (CALI)

**DOI:** 10.1093/ofid/ofac492.043

**Published:** 2022-12-15

**Authors:** Rebecca Osborn, Michael G Ison, Maha M Alamri

**Affiliations:** Northwestern Memorial Hospital, Chicago, Illinois; Northwestern University Feinberg School of Medicine, CHICAGO, Illinois; Northwestern Memorial Hospital, Chicago, Illinois

## Abstract

**Background:**

More than 200 orthotopic lung transplants (OLT) have been performed in the United States for patients (pts) with severe CALI. While some outcomes have been reported to generally be good, we conducted this study to describe the infectious outcomes.

**Methods:**

After IRB approval, a retrospective case review was conducted of pts at Northwestern Memorial Hospital who had undergone OLT for CALI between June 1, 2020 and January 31, 2022. Data was collected from our Enterprise Data Warehouse and primary chart review to describe pts demographics and epidemiology of infectious complications.

**Results:**

35 OLTs were included (33 bilateral, 2 single lung; see Table 1). In the two weeks prior to transplant, 65.7% of pts were treated for bacterial, fungal, or mycobacterial infections (Table 2). Post-transplant, 32 (91.4%) of OLTs developed infections (Table 2), with most (28, 80%) developing pneumonia within 30 days of transplant. Five (14.3%) pts had airway complications, 7 (20%) pts required hemodialysis, and 3 (8.6%) pts died within one year of follow-up.

**Conclusion:**

Infections remain a significant cause of morbidity post-OLT for CALI. Enhanced immunosuppression, long ICU stays post-tx, high rates of primary graft dysfunction and deconditioning likely contributed to this high rate of infection. A matched control group is being collected to compare these outcomes to other OLT pts.

**Disclosures:**

**Michael G. Ison, MD MS**, GlaxoSmithKline: Advisor/Consultant|GlaxoSmithKline: Grant/Research Support.

**Table:**
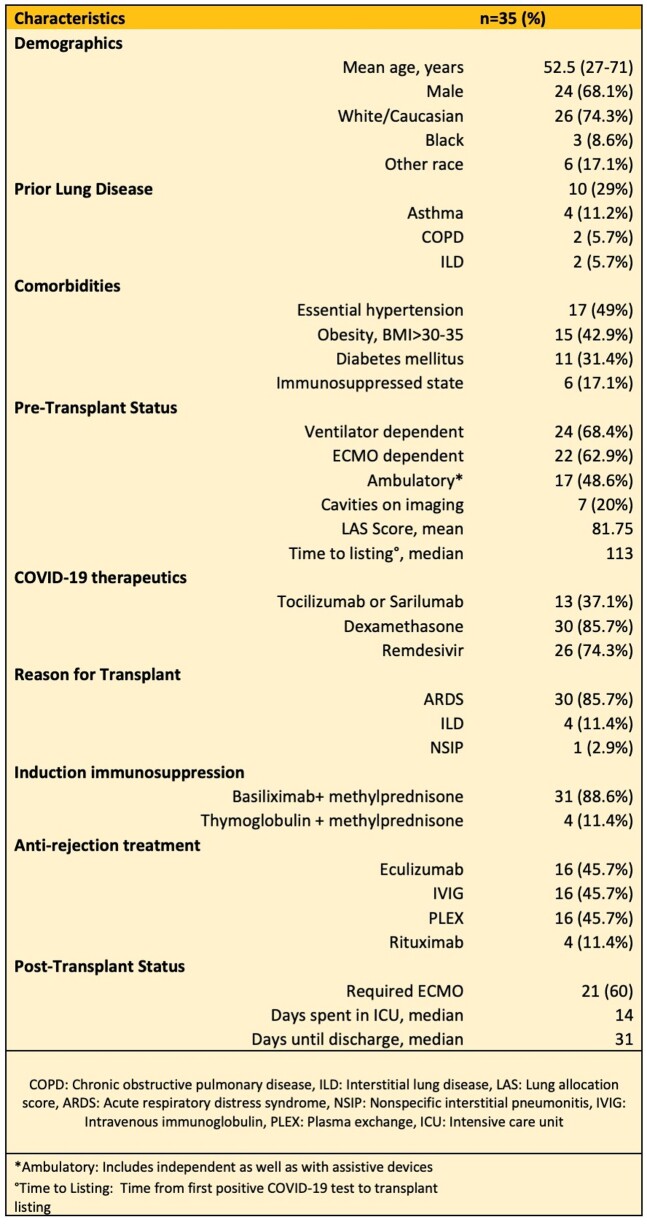
Patient Demographics.

**Table:**
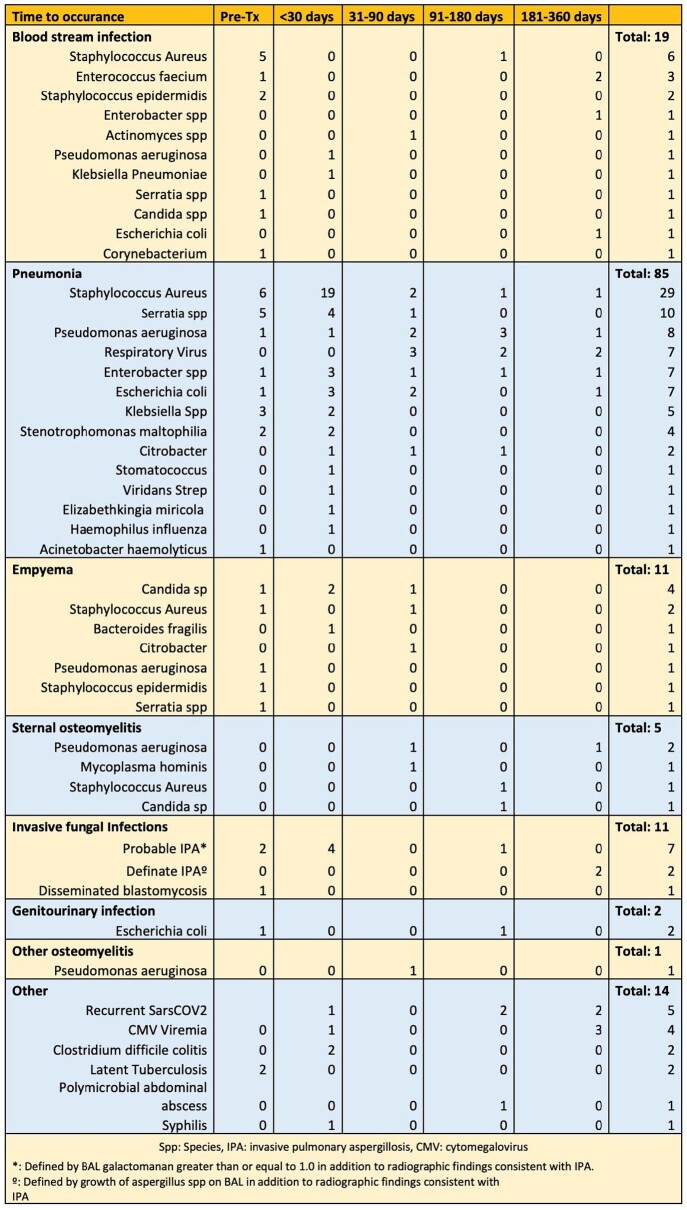
Infectious Complications Post-OLT for CALI.

